# A novel technique of differential lung ventilation in the critical care setting

**DOI:** 10.1186/1756-0500-4-134

**Published:** 2011-05-05

**Authors:** Kazuma Yamakawa, Yasushi Nakamori, Satoshi Fujimi, Hiroshi Ogura, Yasuyuki Kuwagata, Takeshi Shimazu

**Affiliations:** 1 Department of Traumatology and Acute Critical Medicine, Osaka University Graduate School of Medicine, 2-15 Yamadaoka Suita, Osaka 565-0871, Japan; 2 Department of Emergency and Critical Care, Osaka General Medical Center, 3-1-56 Bandai-Higashi, Sumiyoshi-ku, Osaka 558-8558, Japan

## Abstract

**Background:**

Differential lung ventilation (DLV) is used to salvage ventilatory support in severe unilateral lung disease in the critical care setting. However, DLV with a double-lumen tube is associated with serious complications such as tube displacement during ventilatory management. Thus, long-term ventilatory management with this method may be associated with high risk of respiratory incidents in the critical care setting.

**Findings:**

We devised a novel DLV technique using two single-lumen tubes and applied it to five patients, two with severe unilateral pneumonia and three with thoracic trauma, in a critical care setting. In this novel technique, we perform the usual tracheotomy and insert two single-lumen tubes under bronchoscopic guidance into the main bronchus of each lung. We tie the two single-lumen tubes together and suture them directly to the skin. The described technique was successfully performed in all five patients. Pulmonary oxygenation improved rapidly after DLV induction in all cases, and the three patients with thoracic trauma were managed by DLV without undergoing surgery. Tube displacement was not observed during DLV management. No airway complications occured in either the acute or late phase regardless of the length of DLV management (range 2-23 days).

**Conclusions:**

This novel DLV technique appears to be efficacious and safe in the critical care setting.

## Background

Differential lung ventilation (DLV) was reported for the first time in 1931 as a technique for use in thoracic anesthesia [1]. Since then, DLV has been used to salvage ventilatory support in patients with severe unilateral lung disease in the critical care setting [2,3]. The indications for DLV in the critical care setting have not yet been thoroughly established compared to those in thoracic anesthesia [4]. Although Valverde et al. reported that DLV may be a safe technique with few complications [5], displacement of the double-lumen tube during DLV management reportedly occurs in up to 32% of cases when the patient's position is changed [6]. Thus, long-term ventilatory management with this method may be associated with high risk of respiratory incidents in the critical care setting. We devised a novel DLV technique using two single-lumen tubes and evaluated the efficacy and safety of this technique in a critical care setting.

## Materials and methods

A novel DLV technique was applied to five patients with severe hypoxemia refractory to conventional ventilation and application of positive end-expiratory pressure. Two patients had severe unilateral pneumonia, and the other three patients suffered thoracic trauma: one with unilateral pneumothorax and massive air leakage, one with massive hemoptysis due to pulmonary contusion, and one with tracheal rupture at the carina.

This study followed the principles of the Declaration of Helsinki. The ethics committee at our institution does not require its approval or informed consent for retrospective studies such as this study.

**Figure 1:**
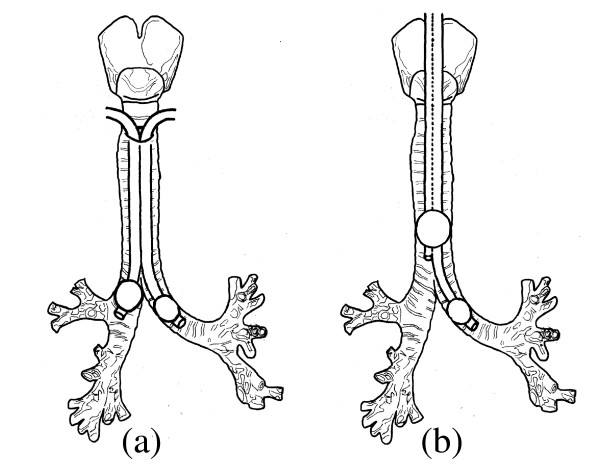
**Schema of two techniques of DLV**. (a) Novel technique using two single-lumen tubes and (b) conventional technique using a double-lumen tube.

**Figure 2:**
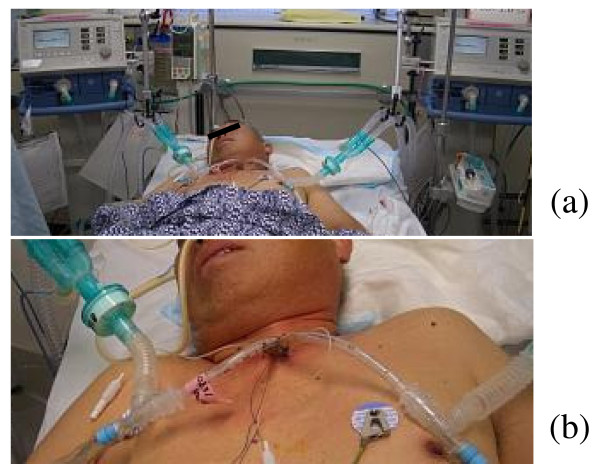
**Photographs showing our DLV technique**. (a, b) Two single-lumen tubes are inserted into the tracheotomy site and are connected to two ventilator circuits respectively.

**Technique **(illustrated in Figures 1 and 2)

In our novel technique, a commercially available double-lumen tube is not used.

1. We perform the usual tracheotomy.

2. We insert a single-lumen tube (7.5-mm ID) under bronchoscopic guidance into the main bronchus of the unaffected lung.

3. We then extend the tracheotomy site using two blunt hooks to secure space for insertion of the second tube. We quickly but carefully insert a second single-lumen tube (7.0-mm ID) under bronchoscopic guidance into the main bronchus of the affected lung.

4. We ascertain proper anatomical position of both tubes by bronchoscopy and chest X-ray.

5. Finally, we tie the two single-lumen tubes together with silk suture and suture them directly to the skin.

6. Differential ventilator settings using two ventilator circuits are applied to each lung asynchronously. As a rule, administration of neuromuscular blockade is not necessary.

## Results

The demographic and clinical details of the five patients undergoing this novel DLV technique are shown in Table 1. The tracheotomy was performed at the time of DLV induction except for one case; in two patients on the day of admission, and in two patients on the day after admission. In the remaining patient, the tracheotomy was performed on the day 5 following admission, and DLV was inducted on the day 20 following admission. Pulmonary oxygenation improved rapidly after DLV induction in all patients. In the three patients with thoracic trauma, massive air leakage or massive hemoptysis disappeared after several days of DLV therapy, and all three patients were managed without undergoing surgery.

**Table 1:** Characteristics of the Patients Undergoing our DLV Technique

	Age	Sex	Clinical Diagnosis	Length of DLV (days)	Length of mechanical ventilation (days)	Length of Follow-up (days)	Outcome
1	65	M	Pneumonia	6	12	33	Survived
2	40	M	Pneumonia	2	21	-	Died
3	73	M	Traumatic pneumothrax	4	25	133	Survived
4	38	M	Pulmonary contusion	5	18	195	Survived
5	25	M	Tracheal rupture of carina	23	26	18	Survived

DLV, differential lung ventilation.

The described technique was successfully performed in all five patients. Complications such as tube displacement, tube obstruction, and tracheal or bronchial injury were not observed regardless of the length of DLV management (range 2-23 days). One patient with unilateral pneumonia died due to septic shock on day 2 of DLV. The four remaining patients were followed up after the end of DLV management, and none experienced complications of airway stenosis after DLV.

## Discussion

Our DLV technique allowed successful ventilatory management of all five patients without complications. These results demonstrate that this novel technique may be effective for ventilatory support in critically ill patients requiring DLV management.

Patient 5 presented with tension pneumopericardium, bilateral pneumothorax, and massive subcutaneous emphysema due to carinal laceration [7]. Operative repair of the tracheal injury could not be performed because of the patient's severe respiratory insufficiency and coagulopathy. Thus, we chose to treat the patient non-operatively, and the initial use of our DLV technique was in this patient. Successful treatment of this patient indicated that our technique could be efficacious in the critical care setting. Here, we examine the efficacy and safety of this technique after its application in five patients.

Although the double-lumen tube of the conventional DLV technique is mainly used in the intensive care setting, serious complications such as tube displacement have been described [6]. Accordingly, there is the need for deep sedation and sometimes neuromuscular blockers for patients on DLV using the double-lumen tube to prevent tube displacement by patient movement or coughing. However, the routine use of neuromuscular blockade is not recommended in mechanically ventilated patients because of complications associated with its use [8,9].

Our DLV technique has several advantages over the conventional DLV method using a double-lumen tube.

1) It is difficult for tube displacement to occur with our method. In case of proximal displacement, it is difficult for bilateral ventilation to occur, unlike with the double-lumen tube, because the cuffs of the two single-lumen tubes prevent their movement above the carina. In case of distal displacement, unilateral ventilation will never occur, unlike with the double-lumen tube, because the distal end of each tube is located within a main bronchus. In addition, there is little influence from head movements because tube fixation is by direct suture at the tracheotomy site [10]. A major reason for the difficulty in displacing the tubes is the firm suturing of each tube to the skin. Our technique allows frequent change of patient position and suctioning of secretions, and administration of neuromuscular blockade is unnecessary to prevent tube displacement.

2) Because the internal diameter of each tube used in our technique is larger than the individual tube diameters in the double-lumen tube, a regular bronchoscope and suction tubing can be used. In addition, risk of tube occlusion is reduced.

3) The two cuffs used in our technique doubly isolate the relatively healthy lung from harmful contaminants in the contralateral diseased lung, whereas with the double-lumen tube, the diseased lung is isolated from the contralateral lung by only one cuff.

4) Ours is the only technique to provide non-operative management of carinal laceration. The advantages of safety and efficacy offered by our DLV technique would be especially appreciated in the critical care setting rather than during surgical anesthesia because of the potentially long period of DLV use generally required in the critical care setting.

Because the performance of tracheotomy is a burden for the patient, the use of our DLV technique should be limited to the rescue of the patients with life-threatening respiratory conditions.

We acknowledge several limitations in this study. First, the design of this study was a case series, and there was no control. The influence of different pathophysiologic issues, such as severity of respiratory failure, lung pathology, and cause of admission to the intensive care unit, on the safety and efficacy of our DLV technique require further evaluation. Second, the sample size in this study was small, and the follow-up period was short. Although we experienced no technical difficulties in insertion of the two tubes or airway complications in either the acute or late phase, further study is required to evaluate the safety of this technique, especially in patients with a narrow trachea such as women and adolescents.

## Conclusion

Our novel DLV technique using two single-lumen tubes may have several advantages of safety and efficacy over the conventional double-lumen tube during the long period of DLV use in the critical care setting.

## Competing interests

The authors declare that they have no competing interests.

## Authors' contributions

KY participated in study design and in data collection and interpretation and drafted the manuscript. YN conceived the study and its design and helped to draft the manuscript. SF, YK, and TS participated in data interpretation. HO had a major impact on the interpretation of data and critical appraisal of the manuscript. All authors read and approved the final manuscript.
